# Transmission of *Cytauxzoon felis* to domestic cats by *Amblyomma americanum* nymphs

**DOI:** 10.1186/s13071-018-3276-8

**Published:** 2019-01-11

**Authors:** Kelly E. Allen, Jennifer E. Thomas, Megan L. Wohltjen, Mason V. Reichard

**Affiliations:** Department of Veterinary Pathobiology, Oklahoma State University Center for Veterinary Health Sciences, Stillwater, OK 74078 USA

**Keywords:** *Amblyomma americanum*, *Cytauxzoon felis*, *Dermacentor variabilis*, Ecdysis, Larvae, Nymphs, Piroplasm, Schizont, Sporozoite, Transstadial

## Abstract

**Background:**

Successful *Cytauxzoon felis* transmission studies have occurred using *Amblyomma americanum* adults acquisition-fed as nymphs on an experimentally infected domestic cat or *Dermacentor variabilis* adults fed as nymphs on a splenectomized bobcat. Here, we evaluated *A. americanum* and *D. variabilis* nymphs acquisition-fed as larvae on a *C. felis*-infected carrier domestic cat for competence to transmit the protozoan parasite as nymphs to naïve, healthy domestic cats.

**Methods:**

*Amblyomma americanum* and *D. variabilis* larvae were applied to a chronically infected, parasitemic *C. felis* donor cat (*Felis catus*) and allowed to feed to repletion. Engorged larvae were collected and held through ecdysis. Three cats were each infested with 66 *A. americanum* or 66 *D. variabilis* emerged nymphs. *Cytauxzoon felis* infections in principal cats were determined by clinical signs and detection of circulating parasite by blood smear and PCR evaluation.

**Results:**

Clinical signs of cytauxzoonosis were observed in cats infested with *A. americanum* nymphs beginning 12–15 days post-infestation (dpi). The same cats were PCR positive on 12–14 dpi; piroplasms were evident in blood smears at 16 dpi, and macrophage schizonts were observed in stained spleen impression smears in two animals at necropsy. Cats infested with acquisition-fed *D. variabilis* nymphs remained clinically normal and did not develop detectable parasitemia over the course of the study as determined by blood smear and PCR.

**Conclusions:**

*Cytauxzoon felis* was successfully transmitted to domestic cats by *A. americanum* nymphs acquisition-fed as larvae on the donor cat. However, we were not able to transmit *C. felis* to healthy domestic cats with *D. variabilis* nymphs that were simultaneously acquisition-fed on the same donor cat. Results from this study suggest that larval and nymphal *A. americanum* likely play important roles in natural transmission cycles of *C. felis*.

## Background

*Cytauxzoon felis* is a blood-borne apicomplexan infecting domestic and wild felids in the southeastern, south-central, and mid-Atlantic USA [1–3]. The disease associated with *C. felis* infection in domestic cats, cytauxzoonosis or bobcat fever, is severe. Salient clinical features of the acute syndrome include an abrupt onset of pyrexia, listlessness, anemia, inappetence, icterus, and dyspnea, with death usually occurring approximately one week after presentation [2, 4, 5]. Survivor domestic cats have been documented and are thought to remain persistently infected with piroplasms in circulation [4, 6–8].

The natural route of *C. felis* transmission to felid intermediate hosts occurs *via* the blood-feeding of infected ticks harboring parasite sporozoites [9, 10]. *Dermacentor variabilis* was initially accepted to be the natural tick vector of *C. felis* based on its presence in some enzootic areas and experiments using splenectomized wild felids [10–13]. More recent experiments documented repeat transstadial transmission of *C. felis* from survivor domestic cats to naïve, immunocompetent cats by *Amblyomma americanum* [9, 10].

In studies surveying wild-caught *A. americanum*, molecular evidence of *C. felis* infection has been detected in 0.0%, 1.5%, or 15.3% of adult ticks sampled and in 0.8% of nymphs sampled [1, 9, 14]. Despite undetectable to low infection prevalence in field-collected *A. americanum* populations in some areas, confirmed experimental data as well as geographical and seasonal trends of cytauxzoonosis cases point to *A. americanum* as the primary definitive host and tick vector of *C. felis* [3, 9, 10].

Previous *C. felis* transmission experiments have utilized adult ticks acquisition-fed as nymphs on donor felids [9, 10, 12, 13]. The present study evaluated the ability of *A. americanum* and *D. variabilis* larvae to acquire *C. felis* from an experimentally infected, parasitemic donor cat and transmit the parasite as nymphs to naïve healthy cats as determined by clinical signs and parasitemia in infested principals.

## Methods

### Animals and housing

Cats were housed individually in cages and maintained indoors in a climate-controlled room with a 12-h light: 12-h dark photocycle. Animals were provided food and water *ad libitum*.

The *C. felis* donor cat was a cytauxzoonosis survivor animal from a previous tick transmission study [9] and was used to infect larval ticks. Six domestic cats (Aa-1, Aa-2, Aa-3, Dv-1, Dv-2 and Dv-3) served as principals for *C. felis* transmission by nymphs acquisition-fed as larvae. All principals were approximately six months of age and weighed between 2.5–3.2 kg at the time of arrival. Cats were vaccinated against feline rhinotracheitis virus, calicivirus, panleukopenia virus, and rabies virus prior to arrival.

During tick infestation experiments, cats were housed in metabolic cages over metal catch pans of water lined along the edges with double-sided tape in a sealed, climate-controlled room at OSU’s Center for Veterinary Health Sciences (Stillwater, OK, USA) [9, 10].

### Acquisition feeding of tick larvae on the *C. felis* donor cat

The donor cat was infested with *A. americanum* and *D. variabilis* larvae hatched from approximately one-half of an egg clutch from females of each species purchased from the National Tick Rearing Lab at Oklahoma State University (Stillwater, OK, USA). Tick species propagated in this laboratory are considered pathogen-free and are therefore widely used by researchers conducting transmission experiments [10, 15].

Prior to infestation, whole blood in EDTA was collected from the *C. felis* donor cat to confirm the presence of piroplasms in erythrocytes in a Wright-Giemsa stained blood film. The donor cat was anesthetized immediately prior to infestation *via* intramuscular injection with 4.0 mg/kg tiletamine and zolazepam (Telazol®; Zoetis, Parsippany, NJ, USA). Larval ticks were applied beneath a Surgi-Sox (DogLeggs, Reston, VA, USA) wrap that covered the donor cat’s thorax and abdomen. The metal catch pan directly beneath the cage and the water moat below were checked daily for replete larvae. PCR was not performed on individual or pooled replete tick larvae, as positive results would have been attributable to *C. felis* piroplasms imbibed from the donor cat, rather than established parasite infections within removed ticks.

### Tick maintenance through ecdysis

Replete larvae acquisition-fed on the *C. felis* donor cat were collected daily and placed in paper cartons that were stored long-term in a humidity chamber (90–98% constant humidity) at 25 °C with a 14-h light-dark photophase. Larvae were held through ecdysis, and emerged nymphs were used to infest separate principal cats in the *C. felis* transmission experiments approximately four to six weeks after molting.

Prevalence of *C. felis* infection in emerged *A. americanum* or *D. variabilis* cohorts was not determined by PCR prior to infestation of principal cats in an effort to preserve potentially infected ticks for transmission experiments. Also, the number of organisms present in the molted nymphs may have been below the level of detection by the PCR assay utilized, even with multiple nymphs being pooled into small groups.

### *Cytauxzoon felis* transmission with emerged nymphs

To confirm that principal cats were not infected with *C. felis* prior to study commencement, whole blood was evaluated for circulating parasite by blood smear examination and PCR. Two separate experiments were conducted to assess *C. felis* transmission to cats using *D. variabilis* and *A. americanum* nymphs that were acquisition-fed as larvae on the donor cat. The numbers of molted nymphs used in the transmission experiments were divided such that each principal within a group (*A. americanum vs D. variabilis*) would receive an equal number of ticks. Nymphs of each species (*n* = 66) were applied to separate naïve cats and allowed to feed to repletion.

In the first transmission experiment, cats Aa-1 and Aa-2 were infested with *A. americanum* nymphs and cats Dv-1 and Dv-2 were infested with *D. variabilis* nymphs. In the second experiment, cat Aa-3 was infested with *A. americanum* nymphs and cat Dv-3 was infested with *D. variabilis* nymphs. Nymphs fed to repletion on cat principals were collected and stored at -20 °C in ethanol for later DNA extraction and PCR analysis.

### Determination of *C. felis* infection in principal cats

Principal cats were observed daily for clinical signs of cytauxzoonosis by physical examination, rectal temperatures, and weights. Additionally, whole blood in EDTA was aseptically collected *via* venipuncture of jugular or cephalic veins from cats at various intervals throughout the study (0, 2, 5, 7, 8, 9, 10, 12, 14 and 16 days post-infestation) for microscopic examination of Wright-Giemsa stained blood films and DNA extraction for PCR.

Cats with *C. felis* parasitemia were euthanatized *via* intravenous administration of Beuthanasia-D (0.20 ml/kg; Schering-Plough, Summit, NJ, USA) after piroplasms were detected in blood and/or after they began showing clinical signs consistent with cytauxzoonosis such as fever, anorexia and lethargy. Cats that did not become infected with *C. felis* were euthanatized or transferred to another protocol.

### DNA extraction and PCR analysis

Total genomic DNA was isolated from 200 μl of whole blood using the QIAamp® DNA Mini Kit (Qiagen, Valencia, CA, USA) per the manufacturer’s instructions. Separate pools of five *A. americanum* and *D. variabilis* replete nymphs that had been stored at -20 °C in ethanol were extracted for DNA as previously described [9, 10].

Primers *C. felis* I/II forward and *C. felis* II reverse and nested primers CfnestF and CfnestR were used to amplify a 289 bp region of the *18S* rRNA gene of *C. felis* from blood and tick extracts [9, 10]. PCR products were separated in a 1.5% agarose matrix stained with ethidium bromide and viewed with ultraviolet light.

## Results

### Confirmation of *C. felis* in donor cat and infestation with larvae

The donor cat was subclinical and had a parasitemia of 0.004% at the time of larvae acquisition feeding as determined by blood film evaluation for piroplasms in erythrocytes. After infestation, as many as possible replete larvae of each tick species were collected (not enumerated) from the donor cat.

### Infestation of principal cats with molted nymphs

The minimum number of acquisition-fed *A. americanum* or *D. variabilis* needed to experimentally transmit *C. felis* to naïve cats is not known, and is likely multi-factorial. In previous experiments, 50–100 *A. americanum* adults and 50–95 *D. variabilis* adults acquisition-fed as nymphs were applied to principal cats; only *A. americanum* infestations resulted in successful *C. felis* infection of principal cats [9, 10, 16]. A total of 230 *A. americanum* and 220 *D. variabilis* molted nymphs were recovered from the donor cat infestation experiment in this study.

Sixty-six molted nymphs were applied to each principal cat in the first transmission experiment; 29 and 9 replete *A. americanum* nymphs were collected from cats Aa-1 and Aa-2, respectively, and 23 and 32 replete *D. variabilis* nymphs  were collected from cats Dv-1 and Dv-2, respectively. In the second experiment, 22 replete *A. americanum* and 22 replete *D. variabilis* nymphs were recovered from cats Aa-3 and Dv-3, respectively. Ticks applied during the infestations but not accounted for at times of collection presumably escaped from Surgi-Sox wraps on principal cats and were groomed off or were trapped in double-sided tape or water moats.

### Clinical signs of *C. felis* infection in principal cats

Cats infested with acquisition-fed *A. americanum* nymphs (Aa-1, Aa-2 and Aa-3) developed clinical signs consistent with cytauxzoonosis, whereas cats infested with acquisition-fed *D. variabilis* nymphs (Dv-1, Dv-2 and Dv-3) did not exhibit clinical signs of cytauxzoonosis at any point during the study. Cat Aa-1 had an elevated body temperature beginning on 14 dpi infestation. Lethargy was observed on 15 dpi and enlarged popliteal lymph nodes were detected on exam on 16 dpi. Cat Aa-2 began losing weight at 12 dpi and maintaining an elevated body temperature on 13 dpi. This cat was observed to be lethargic at 15 dpi and slight splenomegaly was evident upon abdominal palpation the following day. Cat Aa-3 was consistently pyrexic and losing weight beginning 12 dpi and was noted to be depressed and lethargic on 14 dpi. Splenomegaly and hepatomegaly was noted at 15 dpi, and the animal was euthanatized shortly thereafter.

Cats Dv-1 and Dv-2 continued to be monitored by temperature and general health observations until 27 dpi; both cats maintained normal body temperatures and appeared clinically healthy during this time. Cat Dv-3 was monitored for general overall health, was PCR negative on 14 dpi, and was euthanatized at the same time as cat Aa-3.

### Assessment of circulating *C. felis* in principal cats after infestation with nymphs

In previous studies, circulating parasite was detected by PCR and blood smear in experimentally infected cats infested with *A. americanum* by 8–18 dpi and 18 dpi, respectively; time to parasite detection in circulation for cats experimentally infested with *D. variabilis* is unknown, although splenectomized cats died of cytauxzoonosis 13–17 dpi after adult tick engorgement in one study [10, 13, 16].

In the present study, blood sampling of principal cats was ceased once both clinical signs consistent with cytauxzoonosis and observation of parasite on blood films occurred for *A. americanum*-infested cats. Results of blood smear and PCR evaluation of principal cats are summarized in Table 1. Cats Aa-1 and Aa-2 (infested with *A. americanum*) were PCR positive on 12, 14, and 16 dpi; piroplasms were observed in stained blood films at 16 dpi. *Cytauxzoon felis* schizonts were observed in spleen impression smears at the time of necropsy. Cat Aa-3, also infested with *A. americanum* and PCR positive on 14 and 16 dpi, was euthanatized.

**Table 1:** PCR results for *C. felis* infection in principal cats after infestation with *A. americanum* or *D. variabilis* nymphs

Days post-infestation	Cats infested with nymphal *A. americanum*						Cats infested with nymphal *D. variabilis*					
	Cat Aa-1		Cat Aa-2		Cat Aa-3		Cat Dv-1		Cat Dv-2		Cat Dv-3	
	PCR	Smear	PCR	Smear	PCR	Smear	PCR	Smear	PCR	Smear	PCR	Smear
0	–	–	–	–	–	–	–	–	–	–	–	–
2	–	–	–	–	–	–	–	–	–	–	–	–
5	–	–	–	–	ns	ns	–	–	–	–	ns	ns
7	–	–	–	–	ns	ns	–	–	–	–	ns	ns
8	ns	ns	ns	ns	–	–	ns	ns	ns	ns	–	–
9	–	–	–	–	ns	ns	–	–	–	–	ns	ns
10	ns	ns	ns	ns	–	–	ns	ns	ns	ns	–	–
12	+	–	+	–	ns	ns	–	–	–	–	ns	ns
14	+	–	+	–	+	–	–	–	–	–	–	–
16	+	+	+	+	+	–	–	–	–	–	ns	ns

*Abbreviation*: ns, no sample collected or tested

Blood smear and PCR results remained negative for cats infested with *D. variabilis* throughout the study. Although DV-3 was euthanatized after only 16 days of PCR and blood smear monitoring, cats Dv-1 and Dv-2 were monitored by body temperature for 27 dpi, and for general overall health for 35 dpi. On 35 dpi, cat Dv-1 was euthanatized and cat Dv-2 was transferred to another protocol to serve as a negative control animal for *C. felis* cell culture propagation, before which PCR screening of the animal was conducted to confirm lack of *C. felis* infection.

### PCR of replete nymphs for detection of *C. felis* DNA

A single pool of replete nymphs of each tick species was tested for *C. felis* infection by PCR. DNA extracts from separate pools of five replete *A. americanum* and *D. variabilis* nymphs fed on cat principals (held at -20 °C in ethanol) tested negative for *C. felis* DNA. The number of *C. felis* organisms within tick sample pools may have been too low for detection by the PCR method utilized. Xenodiagnostic evidence indicated that a necessary proportion of feeding *A. americanum* were infected with parasite, while this was not the case for feeding *D. variabilis* in this study.

## Discussion

The results of the present study further substantiate *A. americanum* as the predominant natural tick vector in *C. felis* transmission cycles involving domestic cats. Previous studies evaluated *C. felis* transmission to domestic cats using *D. variabilis* and *A. americanum* adults acquisition-fed as nymphs on carrier felids [9, 10, 12, 13]. The present study documented transstadial transmission of *C. felis* in *A. americanum* from larvae to nymphs, and the competence of emerged nymphs to transmit parasite to healthy domestic cats. Nymphal *D. variabilis,* simultaneously acquisition-fed as larvae on the same carrier animal at the time, did not transmit *C. felis* to principal cats.

The inability of *D. variabilis* nymphs to transmit *C. felis* to naïve cats in these experiments may have been influenced by several factors. First, genetic variability of *C. felis* strain internal transcribed spacer (ITS) regions in domestic cats has been documented [2]. The *C. felis* genotype maintained in our experimental donor cats is well-adapted to *A. americanum* based on repeat successful transmission studies with adult ticks, while attempts utilizing *D. variabilis* adults with this strain have failed [9, 10]. Other *C. felis* strains in nature may be more evolutionarily adapted to successful transmission by *D. variabilis*. It is also possible that natural *D. variabilis* populations have a different microbiome than colony reared cohorts which better supports the transstadial maintenance of *C. felis* and subsequent transmission of parasite to felid hosts.

Also, the infection prevalence of *C. felis* between the two tick species could have differed during blood feeding on the donor cat, despite being acquisition-fed on the cat at the same time [9]. *Amblyomma americanum* larvae may have acquired more piroplasms than did *D. variabilis* larvae. As a result, fewer viable sporozoites were present in the salivary glands of molted *D. variabilis* nymphs than in *A. americanum* nymphs, thereby reducing the likelihood of *C. felis* transmission by *D. variabilis.* The relatively low level of parasitemia (0.004% of erythrocytes with piroplasms) in the experimentally infected carrier cat may have also influenced the numbers of viable sporozoites available within ticks after molting. *Dermacentor variabilis* larvae may require a higher number of *C. felis* piroplasms relative to *A. americanum* to maintain enough parasites through the molt to transmit sporozoites during the nymphal stage. As a result, more *D. variabilis* nymphs than *A. americanum* may be needed to successfully transmit *C. felis* to naïve, immunocompetent cats. Time from nymphal emergence to next blood-meal acquisition may also differently affect *C. felis* maintenance and subsequent salivarian transmission between the two tick species.

Previous successful experiments with *D. variabilis* used 400 adults to transmit *C. felis* to immunosuppressed bobcats [12]. Successful experiments with *A. americanum* have been performed using 25 pairs of adult males and females to immunocompetent domestic cats [9, 10]. Here, as few as 33 *A. americanum* nymphs were able to transmit *C. felis* to healthy cats, although not all ticks applied may have been infected; *C. felis* transmission by the same number of *D. variabilis* nymphs did not occur based on absence of both clinical signs and detectable parasitemia in infested cat principals.

We did not detect *C. felis* DNA in nymphal pool DNA extracts of either tick species, but it is possible that the *C. felis* infection prevalence of feeding nymphal cohorts was low, and because of this the few replete ticks randomly pooled for testing were not infected with parasite. It is also possible that organism number between pooled ticks may have been below the detection limit of the PCR assay utilized, especially considering the low level of parasitemia in the donor cat upon which the ticks fed during the previous instar.

In field studies, pools of wild-caught adult *D. variabilis* and *A. americanum* nymphs and adults have tested positive for *C. felis* DNA by PCR, but infection prevalence of *C. felis* in either tick species in nature is highly variable and may be influenced by geographical region. Surveys by different research groups investigating *C. felis* infection prevalence in adult ticks indicate molecular evidence of infection in 0–15.4% of *A. americanum* sampled and in 0–15.9% of *D. variabilis* sampled. C*ytauxzoon felis* has been detected by PCR in 0–0.8% of wild-caught *A. americanum* nymphs surveyed; parasite prevalence in wild-caught *D. variabilis* nymphs has not been documented [9, 17–19]. In these instances, PCR-positive ticks collected in the field may have recently fed on parasitemic hosts, or ticks could have taken a blood meal during previous instars from felid hosts with high enough parasitemia to enable detection of *C. felis* DNA within ticks after ecdysis [9, 20].

Lastly, it is possible that molecular and clinical evidence of *C. felis* infection in cats infested with infected *D. variabilis* may be delayed in comparison to cats infested with infected *A. americanum*. However, although the third clinically normal cat infested with *D. variabilis* nymphs (Dv-3) was euthanatized after only 16 dpi, the first two cats infested with *D. variabilis* nymphs were monitored daily by body temperature for 27 dpi and either euthanatized (Dv-1) or used as a negative control blood source in a cell culture study (Dv-2). The transitioned cat (Dv-2) was screened by PCR to confirm negative *C. felis* status, which it remained. The authors acknowledge that xenodiagnosis may have been a useful approach to further to rule out *C. felis* infection in acquisition-fed ticks, but did not have the opportunity to conduct these experiments.

## Conclusions

**Fig. 1:**
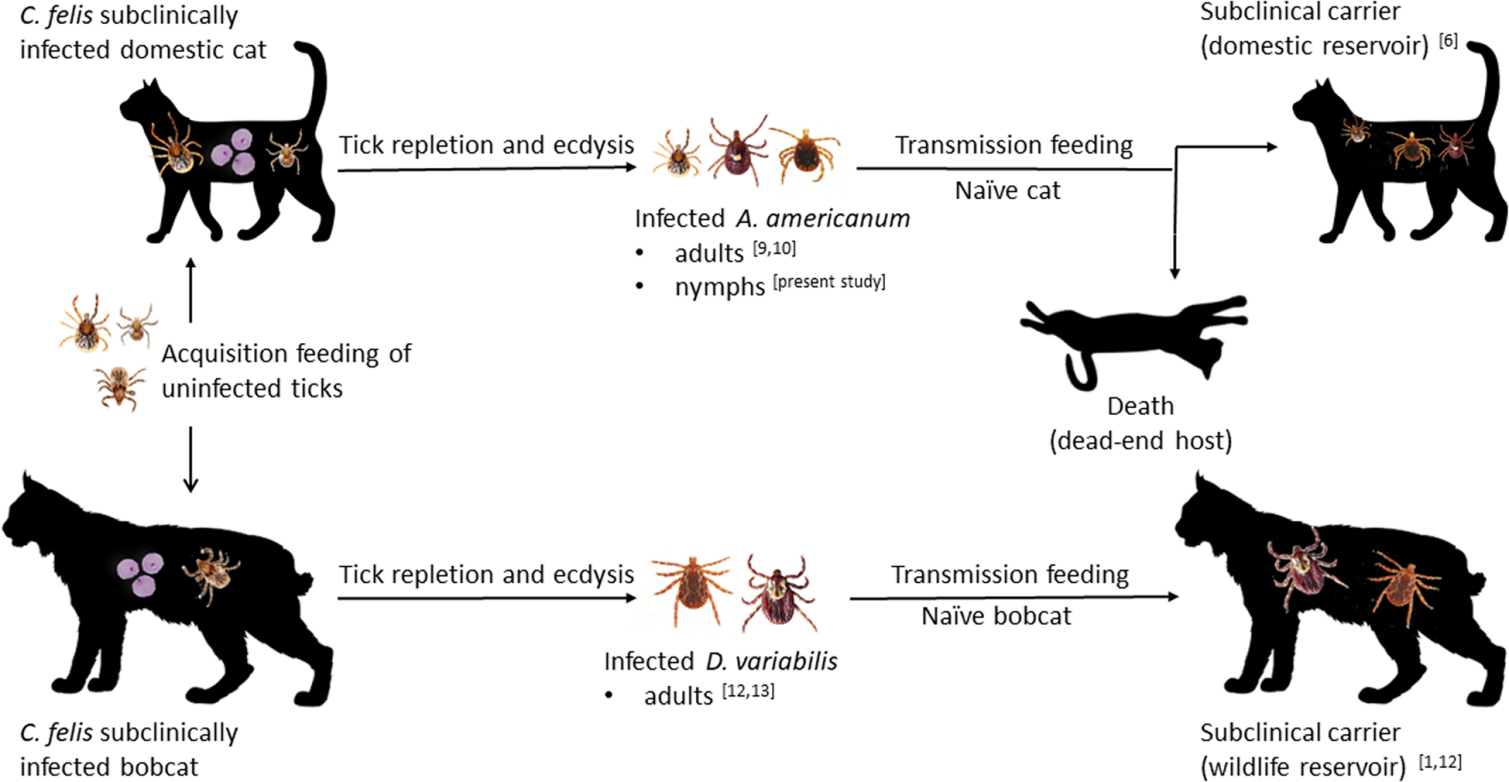
Proposed natural *Cytauxzoon felis* transmission cycles based on experimental data. Transmission of *C. felis* to domestic cats occurs *via* blood-feeding of infected *A. americanum* nymphs and adults acquisition-fed on carrier cats as larvae and nymphs, respectively ([9], present study). Transmission to bobcats occurs *via* blood-feeding of infected *D. variabilis* adults acquisition-fed as nymphs on carrier bobcats [12]. Experimental transmission of *C. felis* by *D. variabilis* to and from domestic cats has not been demonstrated [9, 10]. *Amblyomma americanum* is likely involved in natural *C. felis* cycles in bobcat populations based on overlapping geographical regions of parasitemic bobcats and PCR positive wild-caught ticks [1, 10, 19]

Previously it was shown that feeding *A. americanum* adults, infected with *C. felis* as nymphs, can transmit the parasite to naïve cats. Recent surveys have established that domestic cats are natural hosts of larval, nymphal and adult *A. americanum* [17–19]. Here we demonstrate that feeding *A. americanum* nymphs, infected with *C. felis* as larvae, can transmit the parasite to naïve felid hosts in nature as depicted in Fig. 1. Nymphal *A. americanum* may be an under recognized source of *C. felis* infection to domestic cats.
